# Accuracy of CT Pulmonary Artery Diameter for Pulmonary Hypertension in End-Stage COPD

**DOI:** 10.1007/s00408-016-9926-8

**Published:** 2016-07-16

**Authors:** Firdaus A. Mohamed Hoesein, Tim Besselink, Esther Pompe, Erik-Jan Oudijk, Ed A. de Graaf, J. M. Kwakkel-van Erp, Pim A. de Jong, Bart Luijk

**Affiliations:** 1 Department of Radiology, University Medical Center Utrecht, Heidelberglaan 100, P.O. Box 85500, 3508 Utrecht, The Netherlands; 2 Department of Respiratory Medicine, St. Antonius Hospital, Nieuwegein, The Netherlands; 3 Department of Respiratory Medicine, University Medical Center Utrecht, Utrecht, The Netherlands

**Keywords:** COPD, Pulmonary circulation and pulmonary hypertension, Radiology and other imaging

## Abstract

**Introduction:**

Pulmonary hypertension (PH) in COPD is associated with a higher mortality and an increased risk on exacerbations compared to COPD patients without PH. The aim was to evaluate the diagnostic value of pulmonary artery (PA) measurements on chest computed tomography (CT) for PH in end-stage COPD.

**Methods:**

COPD patients evaluated for eligibility for lung transplantation between 2004 and 2015 were retrospectively analyzed. Clinical characteristics, chest CTs, spirometry, and right-sided heart catheterizations (RHC) were studied. Diameters of PA and ascending aorta (A) were measured on CT. Diagnostic properties of different cut-offs of PA diameter and PA:A ratio in diagnosing PH were calculated.

**Results:**

Of 92 included COPD patients, 30 (32.6 %) had PH at RHC (meanPAP > 25 mm Hg). PA:A > 1 had a negative predictive value (NPV) of 77.9 % and a positive predictive value (PPV) of 63.1 % with an odds ratio (OR (CI 95 %)) of 5.60 (2.00–15.63). PA diameter ≥30 mm had a NPV of 78 % and PPV of 64 % with an OR (CI 95 %) of 6.95 (2.51–19.24).

**Conclusion:**

A small PA diameter and PA:A make the presence of PH unlikely but cannot exclude its presence in end-stage COPD. A large PA diameter and PA:A maybe used to detect PH early.

## Introduction

Chronic obstructive pulmonary disease (COPD) is the fifth leading cause of death worldwide, and estimates show that COPD will become the third leading cause of death in 2030 [1, 2]. Pulmonary hypertension (PH), defined as a mean pulmonary artery (PA) pressure (meanPAP) of >25 mmHg, is a relatively common and important complication in patients with severe COPD [1–4]. PH in COPD is mainly associated with degree of hypoxemia which causes pulmonary vascular constriction and increased precapillary vascular pressure with vascular remodeling. Histopathology demonstrated that the severity of PH correlates with pathologic vascular lesions in pulmonary arteries [5]. PH increases the risk of hospitalization and is associated with a reduced life expectancy [6–8]. Around half of all COPD patients will develop PH and consequently patients with PH tend to have an increased risk on exacerbations, which in turn leads to a greater mortality [9–11]. Moreover, PH is related to a reduced transplant free survival rate [12, 13].

Diagnosing PH remains a challenging clinical problem due to the nonspecific nature of the symptoms. PH-related symptoms like fatigue and exercise-induced dyspnea are often encountered in patients with severe COPD and therefore nonspecific. As a consequence most of the COPD patients are diagnosed with PH by the time the disease already is in an advanced stage [14, 15]. For an adequate treatment early detection is preferable [16, 17]. The gold standard for PH is right heart catheterization (RHC) [17]. However, RHC is invasive, makes use of iodine contrast, and exposes the patient to relatively high radiation doses [17]. A noninvasive tool frequently used in diagnosing PH is echocardiography, but in patients with advanced COPD it showed to be inherently inaccurate due to hyperinflation of the thorax [18, 19].

The routine clinical work-up and follow-up of end-stage COPD patients being screened for lung transplantation includes a chest CT scan. CT scans in end-stage COPD are also often made for a variety of purposes like unexplained acute dyspnea. In this light, a possible diagnostic marker, the PA diameter or the PA to ascending aorta diameter (A) ratio could be used as a noninvasive and opportunistic way to diagnose PH. In COPD patients undergoing CT scanning as routine work-up for lung transplantation, a high negative probability is desired, possibly preventing an invasive RHC. On the other hand, in COPD patients undergoing CT scanning because of any other reason, a high positive predictive value is needed to select those in need of RHC to establish a diagnosis of PH.

Previous studies suggest that PA:A is associated with PH [20–23]. However, there are only few who looked in the diagnostic value of the PA:A on CT in patients with end-stage COPD [24].

Therefore, the purpose of this study is to determine whether the PA:A and PA diameter can be used to diagnose PH in patients with end-stage COPD.

## Methods

### Patient Selection

In this diagnostic accuracy study, we included consecutive patients with a primary diagnosis of COPD or severe emphysema due to α-1-antitrypsin deficiency who were screened for eligibility for potential lung transplantation between 2004 and 2014 at the University Medical Center Utrecht (UMCU). Those with a time interval between the pre transplant chest CT scans and RHC of more than 6 months were excluded. Only patients who underwent both CT scanning and RHC in the UMCU were included, so those with external RCH and/or CT were excluded, resulting in 92 eligible patients of 154 screened in total at the UMCU. Informed consent for this retrospective study was waived by the local institutional review board (IRB), protocol number 14-568/C.

### Right Heart Catheterization

The RHC data collected included mean pulmonary arterial pressure (meanPAP), systolic pulmonary arterial pressure (systPAP), and diastolic pulmonary arterial pressure (diastPAP), and in addition, cardiac output (CO), cardiac index (CI), and pulmonary capillary wedge pressure (PCWP) were evaluated. PH was defined as a meanPAP of 25 mmHg or more [18].

### Clinical Parameters and Pulmonary Function

The following clinical parameters were obtained: sex, age, body mass index (BMI), forced expiratory volume in the first second of exhalation (FEV1) [l], predicted values of FEV1 %, predicted values of total lung capacity (TLC)  %, predicted values of residual volume (RV)  %, packyears smoked, partial oxygen pressure in the blood (Pa02), and outcome from the 6-min walk test (6MWT). COPD was defined by post-bronchodilator spirometry if the FEV1/FVC ratio was below 70 %. The median interquartile range (IQR) number of days between the RHC and CT scan was 42 (8–69).

### CT Protocol and Measurements

**Fig. 1:**
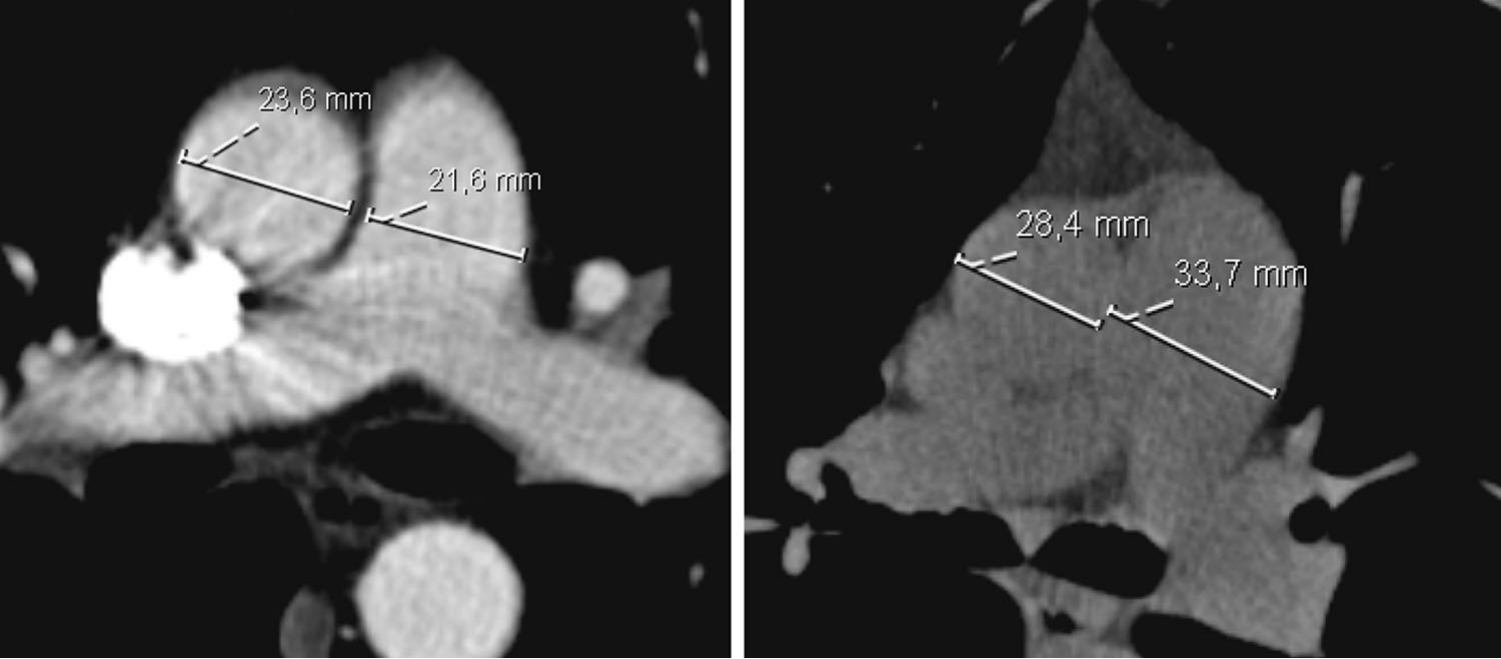
Examples of measurements of the aorta and the pulmonary artery

CT scans were acquired using 16-256 multi-detector row scanners (Philips Medical System), either with or without intravenous contrast. Resolution was below 1 mm. One reviewer (medical student) blinded to all clinical information including the outcome of the RHC analyzed all the CT scans. The PA:A was measured at the level bifurcation of the main PA and within the same slice, the diameter of the ascending aorta was measured (see Fig. 1). The left and right pulmonary arteries were measured directly after the bifurcation. The second blinded reviewer (senior radiology resident) measured the diameters in 25 randomly selected patients in order to assess the interobserver agreement. The intraclass coefficient was excellent 0.85 (CI 95 % 0.69–0.93).

### Statistical Analyses

Data are expressed with mean and standard deviation for normally distributed variables; if the data were not normally distributed, median and IQR were presented.

Unpaired *t* test and Mann–Whitney test were used for continuous variables (normally distributed and not normally distributed data, respectively). For nominal variables, the Chi-squared test was used.

Diagnostic properties to diagnose PH were studied by calculating negative predictive value (NPV), positive predictive value (PPV); sensitivity and specificity were calculated for PA:A, PA diameter, and both the left and right PA diameter. Logistic regression analysis was performed with PH as outcome and PA:A and PA diameter as predictive factors.

## Results

In total, 92 patients were identified with complete datasets without missing CT scans, RHC data, and data within a time frame of 6 months. Baseline characteristics for the total population and split by the presence of PH are displayed in Table 1. Median (IQR) age of the total group was 55.1 (52–59) years and 61 females were included (66.3 %). Mean (SD) FEV_1_ was 0.65 (0.47–0.69) l and the predicted median (IQR) FEV_1_ was 23.1 % (17.0–25.0). Mean (SD) meanPAP was 22.7 (6.6) mmHg, median (IQR) systPAP was 33.6 (28.0–38.8) mmHg, and mean (SD) diastPAP was 15.9 (5.5) mmHg.

**Table 1:** Characteristics for the total population and stratified by absence or presence of pulmonary hypertension (PH)

	No PH *n* = 62	PH *n* = 30	*p* value
Age (IQR) (years)	54.7 year (52–59)	55.9 year (52,75–60)	0.953
Sex (female)	64.5 % female	70.0 % female	0.602
BMI (SD) (kg/m^2^)	23.3 (3.0)	23.4 (2.7)	0.795
Packyears (SD)	31.1 (11.7)	25.8 (10.8)	**0.043**
FEV_1_ (IQR) (l)	0.67 (0.5–0.69)	0.62 L (0.39–0.61)	0.178
Predicted FEV_1_ % (IQR)	23.0 % (27.6–24.6)	23.3 % (15.8–26.5)	0.967
TLC % pred. (SD)	137.2 (20.8)	139.9 (22.3)	0.577
RV % pred.	257.8 (63.2)	274.2 (73.0)	0.286
6MWT (SD) (m)	263.3 (106.3)	229.9 (106.9)	0.058
Change sat (%) during 6 MWT (SD)	10.0 (5.9)	13.3 (7.1)	**0.020**
PaC0_2_	44.8 (7.6)	50.5 (8.3)	**0.002**
Pa0_2_	62.1 (14.3)	56.0 (8.9)	**0.033**
Syst PAP (mmHg)	31.1 (27–35)	39.1 ()	**<0.001**
Diast PAP (SD) (mm Hg)	13.0 (3.8)	21.7 (3.3)	0.102
meanPAP	19.1 (3.6	30.1(5.1)	**<0.001**
CO (L/min)	5.3 (1.3)	5.9 (1.9)	0.075
CI (L/min/m_2_)	3.6 (1.2)	4.2 (1.5)	0.070
PCWP (mmHg)	9.9 (3.5)	13.6 (5.5)	**<0.001**
Aorta (SD) (mm)	30.1 (3.8)	30.8 (3.0)	0.015
Pulmonary artery (SD) (mm)	25.8 (3.2)	29.5 (4.5)	**<0.001**
PA:A (SD)	0.86 (0.12)	0.97 (0.16)	**<0.001**
Left pulmonary (SD) artery (mm)	20.0 (2.6)	21.7 (3.1)	**<0.001**
Right pulmonary (SD) artery (mm)	20.0 (2.7)	20.9 (2.7)	0.24

Bold values indicate statistical significance (*p* < 0.05)

*IQR* Interquartile range, *BMI* body mass index, *TLC* total lung capacity, *RV* residual volume, *6MWT* 6-min walk test, *PaCO2* partial carbon monoxide pressure in the blood, *PaO2* partial oxygen pressure in the blood, *meanPAP* mean pulmonary arterial pressure, *systPAP* systolic pulmonary arterial pressure, *diastPAP* diastolic pulmonary arterial pressure, *CO* cardiac output, *CI* cardiac index, *PCWP* pulmonary capillary wedge pressure, *PA:A* pulmonary artery to aorta ratio

A third of patients (*n* = 30; 32.6 %) had PH (meanPAP ≥ 25 mmHg), of which 2 (2.2 %) suffered from severe PH (meanPAP > 40 mmHg). There were no significant differences in age, sex, FEV_1_, the outcome of the 6MWT, and BMI between patients with and without PH, see Table 1.

Of all included subjects, seven patients had alpha-1-antitrypsin deficiency. There was no significant difference in meanPAP between patients with and without alpha-1-antitrypsin deficiency, 23.7 (11.4) and 22.6 (6.2) mmHg, respectively. Of patients with alpha-1-antitrypsin deficiency, 28.6 % (*n* = 2) had PH compared to 32.9 % (*n* = 28) of patients without alpha-1-antitrypsin deficiency (*p* = 0.588).

Table 2 shows the diameters of the PA, aorta, and the PA:A by the presence of PH. There was a significant difference in PA diameter in subjects with and without PH, 29.5 (4.5) mm and 25.8 (3.2) mm, respectively (*p* < 0.001). Also the PA:A was significantly higher in patients with PH, see Table 1.

**Table 2:** Sensitivity, specificity, positive predictive value (PPV), and negative predictive value (NPV) for different cut-off values of pulmonary artery to aorta ratio (PA:A) in identifying pulmonary hypertension

PA:A	≥0.90	≥0.95	≥1	≥1.05	≥1.10
	*n* = 44	*n* = 34	*n* = 24	*n* = 12	*n* = 8
Sensitivity	0.70	0.60	0.50	0.23	0.17
Specificity	0.63	0.74	0.86	0.94	0.95
PPV	0.48	0.53	0.63	0.64	0.63
NPV	0.81	0.79	0.78	0.72	0.70

**Fig. 2:**
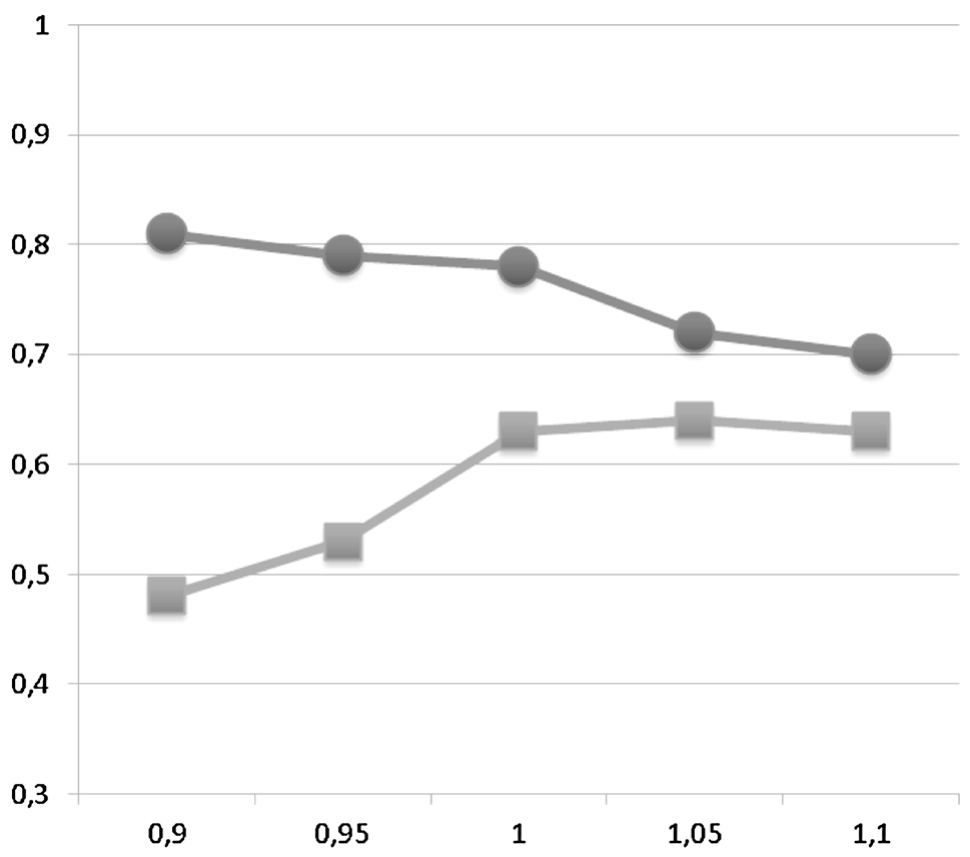
Plot of positive and negative predictive values of different cut-offs for pulmonary artery to aorta ratio (PA:A). The *X*-axis shows the PA:A and the *Y*-axis the responding positive and negative predictive value. The *red line* with *dot* represents the negative predictive value and the *blue line* with *squares* the positive predictive value

**Fig. 3:**
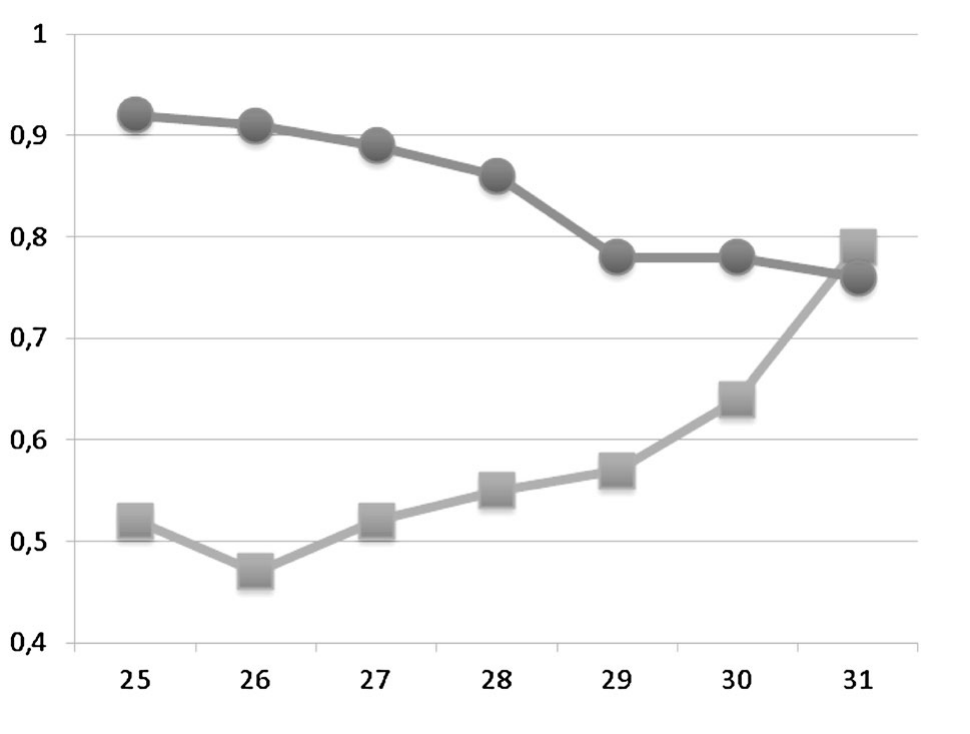
Plot of positive and negative predictive value of different cut-offs for pulmonary artery diameters. The *X*-axis shows the pulmonary artery diameter in millimeters and the *Y*-axis the responding positive and negative predictive value. The *red line* with *dot* represents the negative predictive value and the *blue line* with *squares* the positive predictive value

Sensitivity, specificity and negative and positive predictive values for different cut-offs of PA:A and PA diameter are displayed in Tables 2 and 3, respectively. PPV and NPV are plotted in Figs. 2 and 3 for PAA and PA diameter, respectively. The NPV of a PA:A > 1 was 77.9 % and the PPV was 63.1 %. The sensitivity and specificity were 50.0 and 85.5 %. A PA diameter of >30 mm had a NPV of 78 % and PPV of 57 %. Sensitivity was 47 % and specificity 87 %.

**Table 3:** Sensitivity and specificity for different cut-offs of pulmonary artery (PA) diameter in identifying pulmonary hypertension

PA diameter (mm)	**≥**25	**≥**26	**≥**27	**≥**28	**≥**29	**≥**30	**≥**31
	*n* = 64	*n* = 57	*n* = 48	*n* = 42	*n* = 28	*n* = 22	*n* = 14
Sensitivity	0.90	0.90	0.83	0.77	0.53	0.47	0.37
Specificity	0.60	0.52	0.63	0.69	0.81	0.87	0.95
PPV	0.52	0.47	0.52	0.55	0.57	0.64	0.79
NPV	0.92	0.91	0.89	0.86	0.78	0.78	0.76

Logistic regression analysis showed that per 1 additional mm of PA diameter, the OR (CI 95 %) was 1.31 (1.13–1.51). A PA diameter ≥30 had an OR (CI 95 %) of 5.91 (2. 01–16.58). A PA:A of more than 1 resulted in an OR (CI 95 %) of 5.60 (2.00–15.63).

## Discussion

In this study, we have demonstrated that PA:A and PA diameter measured on chest CT contain substantial diagnostic accuracy for meanPAP at RHC. An increased PA:A and PA diameter on CT may be used as an indicator to raise the clinicians attention for the presence of PH (OR 5), probability of PH > 60 % for PA > 30 mm, and PA:A > 1. A PA diameter <30 mm makes PH unlikely (<78 %); however, CT cannot reliably exclude PH. Therefore, patients with end-stage COPD will still need to undergo RHC if PH needs to be excluded before transplantation.

PH is common in patients with end-stage COPD as found in our study with a prevalence rate of 31.5 %, which is in agreement with percentages found in previous studies and reports reporting a percentage of around 36 % [4]. The relationship of PA:A and PH is of clinical importance as PH is related with an increased mortality and increased exacerbation rate. In earlier stages of COPD, adequate treatment may influence the process of vascular remodeling and improve survival rate and quality of life [25, 26]. Another aspect is the higher complication risk of lung transplant surgery in patients with PH and increased post-transplant mortality [13, 27]. An increased PA:A or large PA diameter on CT may raise the clinicians’ awareness on the possibility that PH is present.

Prior studies have also shown a relationship between PA:A ratio > 1 and PH in COPD, but most data are derived from studies with a heterogeneous group of patients with different types of lung disease [20–23]. Because the pulmonary diameter can differ depending on the underlying lung disease, studies specifically aimed on end-stage COPD are needed. Our study included a well-defined cohort of patients with end-stage COPD. In addition, the hemodynamics and the pathophysiological relationship between COPD and PH may differ for instance from that of PH in sarcoidosis or interstitial pulmonary fibrosis [28].

The findings in the current study are consistent with studies by others. Iyer et al. performed a retrospective study on 60 patients with COPD, and the sensitivity and specificity for a PA:A > 1 were 73 and 84 % for identifying PH [24]. Although, the specificity numbers were similar to our study, sensitivity numbers were higher (in our study the sensitivity was 50 %). This difference could not be explained by a difference in study population. Shin et al. also retrospectively studied a cohort of 65 COPD patients and showed a sensitivity and specificity of a PA:A > 1 of respectively 50 and 93 %, which is similar to our study [20]. A strong point of our study compared to that of Shin is that we used RHC to diagnose PH instead of using the PA:A > 1 or <1.

A PA:A of more than 1 or PA diameter of ≥30 is associated with the presence of PH in our study and it may be of use to increase the suspicion of PH before RHC is performed. One of the most important advantages of using CT scanning is that it is a noninvasive method and that it is readily available. From our study, it is shown that no other clinical parameters differed between the patients with and without PH. Especially because the symptoms of PH show a great overlap with those of COPD, the PA:A may be the only parameter available to the treating physician giving information on the likelihood of PH before RHC is performed.

In addition, CT might also provide relevant information on the extent of emphysema and airway wall disease, which for instance could be of use in determining treatment effect. Ando et al. showed in a small set of COPD subjects that the automated quantification of emphysema and the extent of small pulmonary vessels is associated with a treatment effect of pulmonary vasodilators [29]. Assessing emphysema on CT might also be used to better understand the reduced aerobic exercise capacity in lung transplants candidates with COPD and PH. Data from Adir et al. suggest that lower aerobic exercise capacity is not associated with presence of PH, but with advanced emphysema [30]. Next to emphysema, CT scanning also can provide information on airway wall thickening. Airway wall thickening seems to be associated with severity of PH in patients with COPD. Results of the study by Dournes et al. showed that airway wall thickening on CT was highly associated with meanPAP even when compared to Pa0_2_ [31]. Together, these studies indicate that CT scanning to determine extent of emphysema and airway wall disease in patients with COPD and PH may provide additional information.

Echocardiography is used frequently as noninvasive test for PH as it may estimate the pulmonary arterial pressure. However, in COPD patients, this is often not achievable because of hyperinflation of the thorax; and in only 60 % of cases, echocardiography can measure the pulmonary arterial pressure [19]. In our study, the diameters of the PA could be measured in all cases even in scans without i.v. contrast. The fact that we included both scans with and without i.v. contrast injection is a strong point, because in daily clinical practice both scans with and without contrast are made. Furthermore, these measurements show good reproducibility and require limited training.

A strong point of our study is the large sample size of 92 patients who underwent RCH, making it the largest study on PA:A and PH in end-stage COPD. Furthermore, all patients included were transplanted or were on the current waiting list, and therefore major co morbidities were excluded like coronary artery disease or congestive heart failure. Our study has some limitations. First, our population consisted only of patients with severe COPD awaiting lung transplantation. This may limit generalizability in patients with less severe COPD, but previous literature suggests that our results maybe generalizable [20]. In addition, our included patients have been selected by screening for a lung transplantation waiting list and comorbidities such as left heart disease were exclusion criteria, which may be associated with the occurrence of PH [5, 32]. Second, a limitation remains the single cohort design and retrospective nature of the study. Future studies in other cohorts should be performed to cross-validate our findings.

In conclusion, our data suggest that the PA:A and the PA diameter as measured on chest CT can be used to make the presence of PH very likely, but they cannot be used to reliably exclude PH in COPD patients that are screened for lung transplantation. Patients with a PA diameter > 30 mm or > PA:A > 1 have a high odds ratio of having PH. However, RHC will still remain the gold standard for diagnosing PH.
